# 4-[(1-Benzyl-1*H*-1,2,3-triazol-4-yl)meth­yl]-2*H*-1,4-benzo­thia­zin-3(4*H*)-one

**DOI:** 10.1107/S1600536814000786

**Published:** 2014-01-18

**Authors:** Nada Kheira Sebbar, Abdelfettah Zerzouf, El Mokhtar Essassi, Mohamed Saadi, Lahcen El Ammari

**Affiliations:** a Laboratoire de Chimie Organique Hétérocyclique URAC 21, Pharmacochimie, Avenue Ibn Battouta, BP 1014, Faculté des Sciences, Université Mohammed V-Agdal, Rabat, Morocco; b Laboratoire de Chimie Organique et Etudes Physico-chimique, ENS Takaddoum, Rabat, Morocco; c Laboratoire de Chimie du Solide Appliquée, Faculté des Sciences, Université Mohammed V-Agdal, Avenue Ibn Battouta, BP 1014, Rabat, Morocco

## Abstract

In the title compound, C_18_H_16_N_4_OS, the six-membered heterocycle of the benzo­thia­zine fragment exhibits a screw-boat conformation. The dihedral angles between the plane through the triazole ring and those through the fused and terminal benzene rings are 76.68 (11) and 71.0 (1)°, respectively; the benzene rings are nearly perpendicular [dihedral angle = 79.6 (1)°]. In the crystal, mol­ecules are linked by C—H⋯N and C—H⋯O inter­actions, forming a three-dimensional network.

## Related literature   

For the biological activity of 1,4-benzo­thia­zin-3-one derivatives, see: Rathore & Kumar (2006 ▶); Baraza­rte *et al.* (2008 ▶); Chia *et al.* (2008 ▶). For related structures, see: Ouzidan *et al.* (2011 ▶); Sebbar *et al.* (2014 ▶). For puckering parameters, see: Cremer & Pople (1975 ▶).
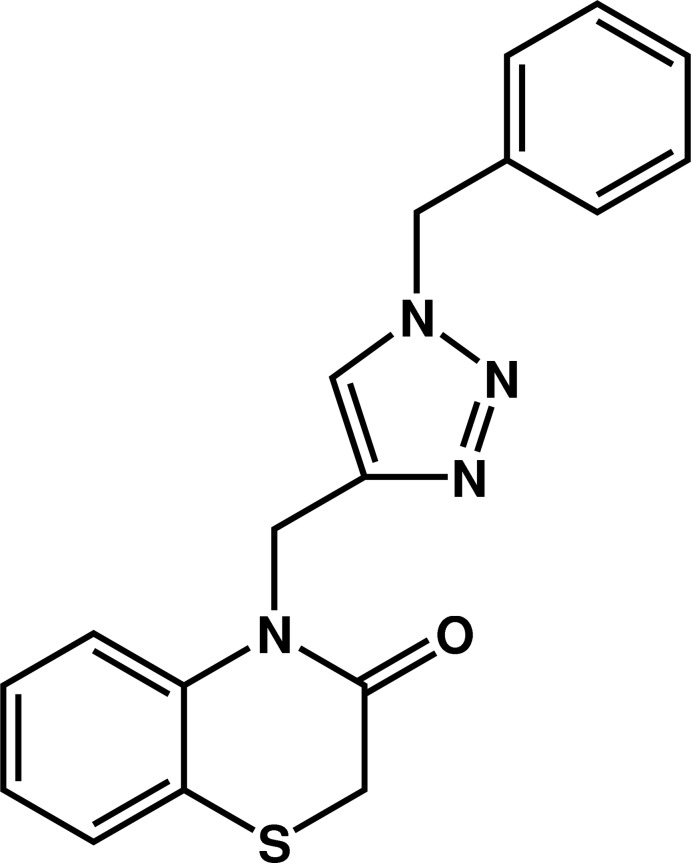


## Experimental   

### 

#### Crystal data  

C_18_H_16_N_4_OS
*M* *_r_* = 336.41
Monoclinic, 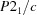

*a* = 13.283 (2) Å
*b* = 5.3661 (10) Å
*c* = 23.281 (4) Å
β = 96.633 (10)°
*V* = 1648.3 (5) Å^3^
*Z* = 4
Mo *K*α radiation
μ = 0.21 mm^−1^
*T* = 296 K
0.39 × 0.35 × 0.28 mm

#### Data collection  

Bruker X8 APEX diffractometer
Absorption correction: multi-scan (*SADABS*; Bruker, 2009 ▶) *T*_min_ = 0.649, *T*_max_ = 0.747
15366 measured reflections
3526 independent reflections
2540 reflections with *I* > 2σ(*I*)
*R*_int_ = 0.044

#### Refinement  

*R*[*F* ^2^ > 2σ(*F* ^2^)] = 0.046
*wR*(*F* ^2^) = 0.149
*S* = 1.03
3526 reflections
217 parameters
H-atom parameters constrained
Δρ_max_ = 0.22 e Å^−3^
Δρ_min_ = −0.33 e Å^−3^

### 

Data collection: *APEX2* (Bruker, 2009 ▶); cell refinement: *SAINT-Plus* (Bruker, 2009 ▶); data reduction: *SAINT-Plus*; program(s) used to solve structure: *SHELXS97* (Sheldrick, 2008 ▶); program(s) used to refine structure: *SHELXL97* (Sheldrick, 2008 ▶); molecular graphics: *ORTEP-3 for Windows* (Farrugia, 2012 ▶); software used to prepare material for publication: *PLATON* (Spek, 2009 ▶) and *publCIF* (Westrip, 2010 ▶).

## Supplementary Material

Crystal structure: contains datablock(s) I. DOI: 10.1107/S1600536814000786/tk5287sup1.cif

Structure factors: contains datablock(s) I. DOI: 10.1107/S1600536814000786/tk5287Isup2.hkl

Supporting information file. DOI: 10.1107/S1600536814000786/tk5287Isup3.cml

CCDC reference:

Additional supporting information:  crystallographic information; 3D view; checkCIF report

## Figures and Tables

**Table 1:** Hydrogen-bond geometry (Å, °)

*D*—H⋯*A*	*D*—H	H⋯*A*	*D*⋯*A*	*D*—H⋯*A*
C8—H8⋯N2^i^	0.93	2.44	3.344 (3)	165
C8—H8⋯N3^i^	0.93	2.44	3.339 (3)	164
C5—H5⋯O1^ii^	0.93	2.58	3.477 (2)	163

Symmetry codes: (i) 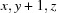; (ii) 
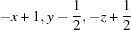
.

**Fig. 1.**
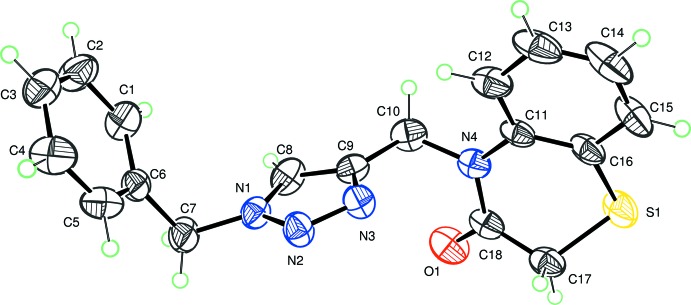
Molecular structure of the title compound with the atom-labelling scheme. Displacement ellipsoids are drawn at the 50% probability level. H atoms are represented as small circles.

**Fig. 2.**
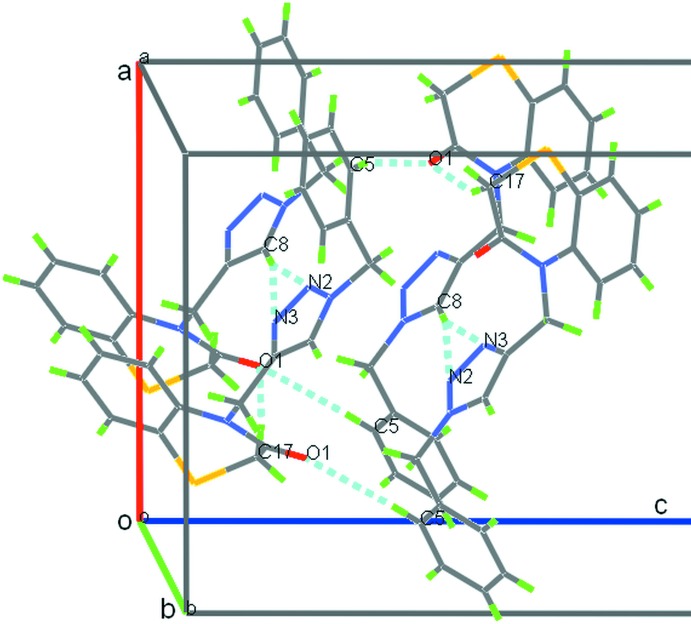
Partial three dimensional plot of the title compound, showing molecules linked through C8–H8···N2, C8–H8···N3 and C5–H5···O1 hydrogen bonds (dashed lines).

## supplementary crystallographic information

### 2.1. Synthesis and crystallization

A mixture of 4-(prop-2-yn-1-yl)-3,4-di­hydro-2*H*-1,4-benzo­thia­zin-3-one (0.27 g, 0.35 mmol) and benzyl­azide (1.34 ml, 0.35 mmol) in ethanol (5 ml) was stirred at room temperature for 24 h. After cooling, the solid obtained was purified by column chromatography on silica gel with ethyl acetate-hexane (1/2) as eluent. Crystals were isolated when the solvent was allowed to evaporate.

### 2.2. Refinement

The H atoms were located in a difference map and treated as riding with C—H = 0.93 Å (aromatic) and C—H = 0.97 Å (methyl­ene), and with *U*_iso_(H) = 1.2 *U*_eq_(C).

### 3. Results and discussion

1,4-Benzo­thia­zine derivatives have a wide spectrum of pharmaceutical and biological activities, such as anti-microbial (Rathore & Kumar, 2006); anti-malarial (Baraza­rte *et al.*, 2008) and anti-inflammatory (Chia *et al.*, 2008). The present work is a continuation of the investigation of the benzo­thia­zine derivatives published recently by our team (Ouzidan *et al.*, 2011; Sebbar *et al.*, 2014). The aim of the present paper was to study the crystal structure of the recently synthesized 4-[(1-benzyl-1*H*-1,2,3-triazol-4-yl)methyl]-3,4-di­hydro-2*H*-1,4-benzo­thia­zin-3-one compound.

The molecule of the title compound is built up from two fused six-membered rings linked to a triazole ring, *via* the heterocyclic, which in turn is attached to benzene ring as shown in Fig. 1. The 1,4-thia­zine ring adopts a screw boat conformation as indicated by the puckering amplitude Q = 0.6197 (17) Å, spherical polar angle θ = 64.42 (16)° and with φ = 329.6 (2)° (Cremer & Pople, 1975). The triazole ring (N1N2N3C8C9) makes dihedral angles of 76.68 (11) and 71.0 (1)° with the benzene fused to the 1,4-thia­zine ring (C11 to C16) and the other benzene ring (C1 to C6), respectively. Moreover, the two benzene rings are nearly perpendicular as indicated by the dihedral angle between them of 79.6 (1)°.

In the crystal, the molecules are linked together by a weak inter­molecular C8–H8···N2, C8–H8···N3 and C5–H5···O1 inter­actions, to form a three-dimensional network (see Fig. 2 and Table 1).

### Crystal data

**Table:**

C_18_H_16_N_4_OS	*F*(000) = 704
*M**_r_* = 336.41	*D*_x_ = 1.356 Mg m^−^^3^
Monoclinic, *P*2_1_/*c*	Mo *K*α radiation, λ = 0.71073 Å
Hall symbol: -P 2ybc	Cell parameters from 3526 reflections
*a* = 13.283 (2) Å	θ = 1.8–27.1°
*b* = 5.3661 (10) Å	µ = 0.21 mm^−^^1^
*c* = 23.281 (4) Å	*T* = 296 K
β = 96.633 (10)°	Block, colourless
*V* = 1648.3 (5) Å^3^	0.39 × 0.35 × 0.28 mm
*Z* = 4	

### Data collection

**Table:**

Bruker X8 APEX diffractometer	3526 independent reflections
Radiation source: fine-focus sealed tube	2540 reflections with *I* > 2σ(*I*)
Graphite monochromator	*R*_int_ = 0.044
φ and ω scans	θ_max_ = 27.1°, θ_min_ = 1.8°
Absorption correction: multi-scan (*SADABS*; Bruker, 2009)	*h* = −16→17
*T*_min_ = 0.649, *T*_max_ = 0.747	*k* = −6→5
15366 measured reflections	*l* = −29→29

### Refinement

**Table:**

Refinement on *F*^2^	Primary atom site location: structure-invariant direct methods
Least-squares matrix: full	Secondary atom site location: difference Fourier map
*R*[*F*^2^ > 2σ(*F*^2^)] = 0.046	Hydrogen site location: inferred from neighbouring sites
*wR*(*F*^2^) = 0.149	H-atom parameters constrained
*S* = 1.03	*w* = 1/[σ^2^(*F*_o_^2^) + (0.087*P*)^2^ + 0.1602*P*] where *P* = (*F*_o_^2^ + 2*F*_c_^2^)/3
3526 reflections	(Δ/σ)_max_ < 0.001
217 parameters	Δρ_max_ = 0.22 e Å^−^^3^
0 restraints	Δρ_min_ = −0.33 e Å^−^^3^

### Special details

**Table:**

Geometry. All e.s.d.'s (except the e.s.d. in the dihedral angle between two l.s. planes) are estimated using the full covariance matrix. The cell e.s.d.'s are taken into account individually in the estimation of e.s.d.'s in distances, angles and torsion angles; correlations between e.s.d.'s in cell parameters are only used when they are defined by crystal symmetry. An approximate (isotropic) treatment of cell e.s.d.'s is used for estimating e.s.d.'s involving l.s. planes.
Refinement. Refinement of *F*^2^ against all reflections. The weighted *R*-factor *wR* and goodness of fit *S* are based on *F*^2^, conventional *R*-factors *R* are based on *F*, with *F* set to zero for negative *F*^2^. The threshold expression of *F*^2^ > σ(*F*^2^) is used only for calculating *R*-factors(gt) *etc*. and is not relevant to the choice of reflections for refinement. *R*-factors based on *F*^2^ are statistically about twice as large as those based on *F*, and *R*- factors based on all data will be even larger.

### Fractional atomic coordinates and isotropic or equivalent isotropic displacement parameters (Å^2^)

**Table:**

	*x*	*y*	*z*	*U*_iso_*/*U*_eq_	
C1	0.22144 (17)	1.2104 (4)	0.32721 (9)	0.0608 (6)	
H1	0.2703	1.3328	0.3361	0.073*	
C2	0.13131 (19)	1.2201 (4)	0.35176 (10)	0.0663 (6)	
H2	0.1207	1.3471	0.3776	0.080*	
C3	0.05772 (18)	1.0436 (4)	0.33828 (10)	0.0620 (6)	
H3	−0.0028	1.0501	0.3547	0.074*	
C4	0.07467 (19)	0.8572 (5)	0.30010 (9)	0.0675 (7)	
H4	0.0246	0.7387	0.2901	0.081*	
C5	0.16506 (19)	0.8438 (4)	0.27650 (8)	0.0577 (6)	
H5	0.1759	0.7140	0.2515	0.069*	
C6	0.23928 (15)	1.0199 (4)	0.28950 (7)	0.0457 (4)	
C7	0.33713 (17)	1.0077 (4)	0.26263 (8)	0.0597 (6)	
H7A	0.3309	0.8829	0.2323	0.072*	
H7B	0.3490	1.1673	0.2450	0.072*	
C8	0.48825 (17)	1.0970 (4)	0.33613 (9)	0.0545 (5)	
H8	0.4885	1.2703	0.3354	0.065*	
C9	0.55342 (15)	0.9457 (3)	0.36961 (7)	0.0424 (4)	
C10	0.64140 (18)	1.0099 (4)	0.41219 (9)	0.0555 (5)	
H10A	0.6711	1.1646	0.4006	0.067*	
H10B	0.6181	1.0363	0.4497	0.067*	
C11	0.72308 (14)	0.6346 (4)	0.46251 (7)	0.0438 (4)	
C12	0.64100 (17)	0.6069 (5)	0.49446 (8)	0.0581 (6)	
H12	0.5823	0.6994	0.4847	0.070*	
C13	0.6465 (2)	0.4417 (5)	0.54082 (8)	0.0727 (8)	
H13	0.5923	0.4296	0.5627	0.087*	
C14	0.7301 (2)	0.2979 (6)	0.55454 (8)	0.0780 (8)	
H14	0.7327	0.1873	0.5854	0.094*	
C15	0.81136 (19)	0.3164 (5)	0.52239 (8)	0.0683 (7)	
H15	0.8680	0.2154	0.5311	0.082*	
C16	0.80820 (15)	0.4867 (4)	0.47686 (7)	0.0499 (5)	
C17	0.84430 (15)	0.5783 (4)	0.36944 (7)	0.0526 (5)	
H17A	0.8910	0.6067	0.3410	0.063*	
H17B	0.8032	0.4341	0.3572	0.063*	
C18	0.77735 (15)	0.8007 (4)	0.37190 (7)	0.0480 (5)	
N1	0.42420 (12)	0.9464 (3)	0.30471 (6)	0.0442 (4)	
N2	0.44742 (13)	0.7078 (3)	0.31697 (6)	0.0482 (4)	
N3	0.52629 (12)	0.7072 (3)	0.35664 (6)	0.0461 (4)	
N4	0.71926 (12)	0.8149 (3)	0.41721 (6)	0.0451 (4)	
O1	0.77092 (14)	0.9600 (3)	0.33403 (6)	0.0689 (5)	
S1	0.91484 (4)	0.51598 (14)	0.43858 (2)	0.0692 (2)	

### Atomic displacement parameters (Å^2^)

**Table:**

	*U*^11^	*U*^22^	*U*^33^	*U*^12^	*U*^13^	*U*^23^
C1	0.0529 (13)	0.0505 (13)	0.0794 (14)	−0.0036 (10)	0.0088 (11)	−0.0073 (10)
C2	0.0640 (15)	0.0553 (13)	0.0817 (14)	0.0067 (11)	0.0168 (12)	−0.0186 (11)
C3	0.0530 (13)	0.0659 (14)	0.0694 (13)	0.0021 (11)	0.0169 (11)	0.0023 (11)
C4	0.0738 (16)	0.0651 (15)	0.0658 (12)	−0.0245 (12)	0.0177 (12)	−0.0075 (11)
C5	0.0784 (16)	0.0501 (12)	0.0461 (9)	−0.0030 (11)	0.0137 (10)	−0.0047 (9)
C6	0.0480 (11)	0.0503 (11)	0.0385 (8)	0.0108 (9)	0.0041 (7)	0.0125 (8)
C7	0.0553 (13)	0.0785 (16)	0.0465 (10)	0.0182 (11)	0.0108 (9)	0.0225 (10)
C8	0.0598 (13)	0.0292 (9)	0.0759 (13)	0.0004 (9)	0.0141 (11)	0.0046 (9)
C9	0.0480 (11)	0.0325 (9)	0.0483 (9)	−0.0044 (8)	0.0125 (8)	−0.0029 (7)
C10	0.0621 (13)	0.0426 (11)	0.0615 (11)	−0.0112 (10)	0.0064 (10)	−0.0110 (9)
C11	0.0433 (10)	0.0563 (11)	0.0312 (7)	−0.0180 (9)	0.0015 (7)	−0.0067 (7)
C12	0.0483 (12)	0.0824 (15)	0.0449 (10)	−0.0193 (11)	0.0101 (9)	−0.0094 (10)
C13	0.0673 (16)	0.114 (2)	0.0379 (9)	−0.0397 (15)	0.0129 (10)	−0.0035 (11)
C14	0.0828 (19)	0.110 (2)	0.0378 (9)	−0.0344 (16)	−0.0084 (10)	0.0178 (11)
C15	0.0655 (15)	0.0909 (18)	0.0437 (10)	−0.0127 (13)	−0.0145 (9)	0.0138 (11)
C16	0.0410 (10)	0.0736 (14)	0.0332 (8)	−0.0165 (10)	−0.0039 (7)	−0.0012 (8)
C17	0.0461 (11)	0.0736 (14)	0.0391 (8)	−0.0139 (10)	0.0094 (8)	−0.0017 (9)
C18	0.0469 (11)	0.0581 (12)	0.0387 (8)	−0.0221 (9)	0.0036 (7)	−0.0004 (8)
N1	0.0450 (9)	0.0419 (9)	0.0469 (8)	0.0054 (7)	0.0102 (7)	0.0093 (6)
N2	0.0531 (10)	0.0375 (9)	0.0513 (8)	0.0015 (7)	−0.0049 (7)	0.0012 (7)
N3	0.0538 (10)	0.0318 (8)	0.0503 (8)	−0.0042 (7)	−0.0035 (7)	−0.0009 (6)
N4	0.0435 (9)	0.0497 (9)	0.0419 (7)	−0.0123 (7)	0.0044 (6)	−0.0033 (7)
O1	0.0803 (12)	0.0684 (10)	0.0588 (8)	−0.0190 (8)	0.0122 (8)	0.0172 (7)
S1	0.0364 (3)	0.1170 (6)	0.0528 (3)	−0.0050 (3)	−0.0005 (2)	0.0036 (3)

### Geometric parameters (Å, º)

**Table:**

C1—C6	1.385 (3)	C10—H10B	0.9700
C1—C2	1.386 (3)	C11—C16	1.390 (3)
C1—H1	0.9300	C11—C12	1.397 (3)
C2—C3	1.371 (3)	C11—N4	1.428 (2)
C2—H2	0.9300	C12—C13	1.392 (3)
C3—C4	1.374 (3)	C12—H12	0.9300
C3—H3	0.9300	C13—C14	1.360 (4)
C4—C5	1.379 (3)	C13—H13	0.9300
C4—H4	0.9300	C14—C15	1.387 (4)
C5—C6	1.374 (3)	C14—H14	0.9300
C5—H5	0.9300	C15—C16	1.396 (3)
C6—C7	1.508 (3)	C15—H15	0.9300
C7—N1	1.464 (3)	C16—S1	1.766 (2)
C7—H7A	0.9700	C17—C18	1.493 (3)
C7—H7B	0.9700	C17—S1	1.798 (2)
C8—N1	1.330 (3)	C17—H17A	0.9700
C8—C9	1.364 (3)	C17—H17B	0.9700
C8—H8	0.9300	C18—O1	1.224 (2)
C9—N3	1.354 (2)	C18—N4	1.379 (2)
C9—C10	1.483 (3)	N1—N2	1.340 (2)
C10—N4	1.466 (3)	N2—N3	1.314 (2)
C10—H10A	0.9700		
			
C6—C1—C2	120.5 (2)	C16—C11—C12	118.32 (18)
C6—C1—H1	119.7	C16—C11—N4	121.53 (16)
C2—C1—H1	119.7	C12—C11—N4	120.14 (19)
C3—C2—C1	120.5 (2)	C13—C12—C11	120.4 (2)
C3—C2—H2	119.8	C13—C12—H12	119.8
C1—C2—H2	119.8	C11—C12—H12	119.8
C2—C3—C4	119.0 (2)	C14—C13—C12	120.8 (2)
C2—C3—H3	120.5	C14—C13—H13	119.6
C4—C3—H3	120.5	C12—C13—H13	119.6
C3—C4—C5	120.7 (2)	C13—C14—C15	119.9 (2)
C3—C4—H4	119.6	C13—C14—H14	120.1
C5—C4—H4	119.6	C15—C14—H14	120.1
C6—C5—C4	120.80 (19)	C14—C15—C16	120.0 (2)
C6—C5—H5	119.6	C14—C15—H15	120.0
C4—C5—H5	119.6	C16—C15—H15	120.0
C5—C6—C1	118.43 (18)	C11—C16—C15	120.55 (19)
C5—C6—C7	120.64 (18)	C11—C16—S1	120.37 (14)
C1—C6—C7	120.92 (19)	C15—C16—S1	119.07 (18)
N1—C7—C6	112.60 (14)	C18—C17—S1	111.42 (13)
N1—C7—H7A	109.1	C18—C17—H17A	109.3
C6—C7—H7A	109.1	S1—C17—H17A	109.3
N1—C7—H7B	109.1	C18—C17—H17B	109.3
C6—C7—H7B	109.1	S1—C17—H17B	109.3
H7A—C7—H7B	107.8	H17A—C17—H17B	108.0
N1—C8—C9	106.02 (17)	O1—C18—N4	120.9 (2)
N1—C8—H8	127.0	O1—C18—C17	121.52 (17)
C9—C8—H8	127.0	N4—C18—C17	117.51 (16)
N3—C9—C8	107.47 (18)	C8—N1—N2	110.29 (16)
N3—C9—C10	122.51 (17)	C8—N1—C7	129.57 (18)
C8—C9—C10	130.00 (18)	N2—N1—C7	120.13 (17)
N4—C10—C9	112.43 (15)	N3—N2—N1	107.31 (15)
N4—C10—H10A	109.1	N2—N3—C9	108.92 (15)
C9—C10—H10A	109.1	C18—N4—C11	123.52 (17)
N4—C10—H10B	109.1	C18—N4—C10	115.48 (16)
C9—C10—H10B	109.1	C11—N4—C10	120.46 (15)
H10A—C10—H10B	107.8	C16—S1—C17	95.91 (9)

### Hydrogen-bond geometry (Å, º)

**Table:**

*D*—H···*A*	*D*—H	H···*A*	*D*···*A*	*D*—H···*A*
C8—H8···N2^i^	0.93	2.44	3.344 (3)	165
C8—H8···N3^i^	0.93	2.44	3.339 (3)	164
C5—H5···O1^ii^	0.93	2.58	3.477 (2)	163

Symmetry codes: (i) *x*, *y*+1, *z*; (ii) −*x*+1, *y*−1/2, −*z*+1/2.
